# Supplementary material for: Clinical and laboratory presentation of first-time antenatal care visits of pregnant women in Ghana, a hospital-based study
Source: PLoS One. 2023 Jan 4;18(1):e0280031. doi: 10.1371/journal.pone.0280031 (PMC9812315; doi:10.1371/journal.pone.0280031)
Supplement: Attachment — Submitted filename: Response to Reviewers.docx [file pone.0280031.s002.docx]

May 15^th^, 2022

PLOS ONE

Dear editor,

Thank you for the opportunity to revise our manuscript titled “Clinical and laboratory presentation of first-time antenatal care visits of pregnant women in Ghana, a hospital-based study” with reference number “PONE-D-21-22935”.

We find the reviewer(s) comments helpful and have therefore revised the manuscript with the reviewer’s comments to the authors. We have provided a response (highlighted) to the comments in the detailed document attached.

Thank you,

Maxwell Akonde

akondemaxwell@gmail.com

**Response to reviewer’s comments**

1. Please ensure that your manuscript meets PLOS ONE's style requirements, including those for file naming. The PLOS ONE style templates can be found at

**Manuscript was formatted to meet PLOS ONE’s style requirement**

<https://journals.plos.org/plosone/s/file?id=wjVg/PLOSOne_formatting_sample_main_body.pdf> and

<https://journals.plos.org/plosone/s/file?id=ba62/PLOSOne_formatting_sample_title_authors_affiliations.pdf>.

2. In the ethics statement in the manuscript and in the online submission form, please provide additional information about the patient records used in your retrospective study, including: a) whether all data were fully anonymized before you accessed them; b) the date range (month and year) during which patients' medical records were accessed; c) the date range (month and year) during which patients whose medical records were selected for this study sought treatment. If the ethics committee waived the need for informed consent, or patients provided informed written consent to have data from their medical records used in research, please include this information.

**We have provided additional information including the information on informed consent, date range the women’s records were accessed and date range of the women during which they sought treatment.**

3. Please include your full ethics statement in the ‘Methods’ section of your manuscript file. In your statement, please include the full name of the IRB or ethics committee who approved or waived your study, as well as whether or not you obtained informed written or verbal consent. If consent was waived for your study, please include this information in your statement as well.

**We have provided full ethics statement in the Methods section**

Reviewers' comments:

**Comments to the Author**

5. Review Comments to the Author

Please use the space provided to explain your answers to the questions above. You may also include additional comments for the author, including concerns about dual publication, research ethics, or publication ethics. (Please upload your review as an attachment if it exceeds 20,000 characters)

Reviewer #1: General comments: The manuscript treats an important question about maternal health in a low-income community.
As the data were collected retrospectively in 2018, It could have been great to add information on the outcomes (mother/offspring) associated with the conditions studied.

Abstract
In general, the abstract will need to be modified after the modification of the main text.

**The abstract was modified, and more details were added to the introduction, methods and conclusion to reflect some changes in the main text**

Line 35: it will be better to have hemoglobin phenotyping after hemoglobin level on line 33

**Hemoglobin phenotyping was place after hemoglobin level**
Line 41- 43: conclusion needs to be improved. The fact that pregnant women were asymptomatic or symptomatic was not stated in the text.

**The conclusion was improved as captured in the revised manuscript**
Conclusion highlights only malaria and anemia.

**Malaria and anemia were the most significant findings and we thinking highlighting these was the most appropriate. We improved the conclusion, however, by adding the need for interventions to be directed at addressing these findings.**

Introduction
The introduction needs to address clearly the research question and the objective of the study

**I have clearly defined the research question and objective of the study in the last paragraph of the introduction.**

Lines 53-55: please reformulate
**I reformulated this sentence and made it more concise and clearer.**
Methodology
There is only little information provided in this section.

**I have provided more and detailed information on this section**
Study area: please provide general information on the background, rural/urban health services available, uptake of antenatal interventions

**I have provided general information on the study area**
The description of the health facility where the data come from is lacking (how many pregnant women attend antenatal care, how many deliveries per month/year?

**I have provided further essential information including average number of antenatal cases and number of days for antenatal clinics per day.**
On average, when do pregnant women start the antenatal care in the region/district? What is/are the main source(s) of income

**Data on when pregnant women attend antenatal care was not available**

Nutritional status of the population (preschool children, pregnant women)
Please provide information on altitude, which can affect the interpretation of hemoglobin.

**The interpretation of hemoglobin level in Ghana, and for that matter the area is based on recommended reference range by World Health Organization**

Line72: is the “weight” demographic parameter or anthropometric/clinical?

**Weight is demographic and has been corrected to reflect that.**
The authors need to provide information on the assays /platforms used to diagnose the conditions studied HIV, HBV… and the interpretation of the results

**We provided more information on the assays for all the laboratory tests that were done and the interpretation of these assays.**

Please provide information on the management of those who were positive for HIV, HVB…

**Infection management was not within the scope of the manuscript and hence we did not include data on management of those who tested positive for HIV, HBV, HCV, syphilis, malaria.**
I guess the variable “Height” may have been recorded, why it is not part of the data?

**The height of the pregnant women was not measured and hence we could not include that in this study**
Gestational age is important and should be integrated

**Information on gestational age was missing/incomplete and hence we could not include that in the current study**
More information about the cut-off used for anemia is needed (reference…)

**A reference has been added**
I suggest presenting a flow chart showing the process of data collection
**This was a retrospective cross-sectional study that utilized a complete case analysis. Hence we do not think a flow chart will add much information to the manuscript.**
Results
General: please use one decimal (line 77, 78,79…)

**We used one decimal throughout the manuscript including the tables.**
Information in tables are redundant with those in the comment accompanying tables

**Table one was collapsed and the information on it was added to table two to avoid redundancy.**
All transformation of variables should be indicated in the methodology section (example age)
**We captured all transformation of variables in the methods section and described all recoding as well.**
Table 1: the title of the table stipulate “Demographic characteristic…” but in the text there are clinical and biological data
It is not important to present the mean and the mode, I suggest removing the mode
**Table 1 was collapsed and the information on table 1 added to table 2.**
Table 2. I suggest changing the title. According to the title of the manuscript table 2 should be looking like “Laboratory characteristics of pregnant women …” (just an example)

**We changed the title of the table to reflect what is captured on the table**
Information in this table needs to be describe in the methodology (meaning, how the diagnosis was made…)

**We presented details of the information including how diagnosis was made in the methods**
Line 93: not clear

**We rephrased this sentence to provide clarity**
Line 95: provide the source of the cut-off and put it the methodology

**This was put in the methods section and a reference was provided**
Lines 96-98:
Table 3: the cut-offs of anemia and not-anemia overlap need to be clarified (<=11.5 and >=11.5)

**We clarified the cut-offs. We used <11.5 and >=11.5 for the analysis and hence corrected the overlap to reflect this.**
I suggest adding two rows: one with all infections combined and the second presenting subject without infection
It doesn’t seem to be important to have P value for HBV

**We agreed with this and removed the P-value for HBV**
Give information on the statistical test(s) used

**Information on the statistics test has been provided in the methods section**
Table 4: the title should be changed, it says demographic characteristics and infections… but there is no infection in the data presented

**The title of the table was changed to reflected to the information on the table.**
Table 4 needs legend with explanation for abbreviations
**A legend was provided to explain the abbreviations**
Discussion
Line 120: …important for the management and prevention (please add prevention)

**Prevention was added**
Lines 128: iron needed for red cells production/ hemoglobin?

**Red cell production was changed to hemoglobin as iron is directly used for hemoglobin synthesis**
Lines 133-134: please confirm micronutrients recommended during pregnancy by WHO

**Micronutrients were confirmed and updated**
Lines 141-142: nutritional status of pregnant women is also affected by the diet before pregnancy

**This was highlighted, even though our study did not include data on nutritional status before pregnancy**
Lines 142-143: Please reformulate the sentence

**The sentence was reformulated to ensure clarity**
Lines 144-145: need also to compare with a country (countries) in the west African region
Lines 150: it is not clear what means “…other malaria preventive techniques…”

**We clarified and rephrased this sentence**
As malaria diagnosis was done per routine protocol, it is likely that microscopy and/or rapid diagnosis test was/were used; sub microscopy (common during pregnancy) needs to be discussed

**The malaria protocol was expanded in the methods section. We emphasized the use of microscopy for confirming the positive RDTs**
Lines 160-161: what is the picture in the general population?
Lines 161-162: what was done to explain the “success story”, what was the previous picture and what is the current in the general population? How representative are these results from one hospital (June-September 2018)?

**We expanded on the “success story”. The lack of representative is one of the limitations we discussed**
Lines 162-163: what could be the explanations.

**We offered an explanation or mechanism involved.**
Lines 164-165: the fact that most of the infections studied are blood-borne doesn’t fit as a possible explanation for infected pregnant women to be more anemic. Please revise the pathogenesis of anemia related to malaria and anemia related to infection other than malaria and also anemia associated with chronic infection/inflammation

**We revised the possible explanation of infection and anemia**
Lines 166-167: it is not clear whether after transfusion women were still anemic

**We had no information on transfusion and so we revised this appropriately**
Line 168: test hypothesis instead of establish hypothesis

**This statement was revised and removed to reflect the changes made**
Line 175: the authors need to add gestational age on their data; I assume this information is available in the record from the hospital

**Information on gestational age was missing or incomplete and hence we could not include that in this study**
Lines 181-183: Which could be the risk factor? Is anemia the risk factor for infections or infections are the risk factors for anemia?

**Infections such as malaria are a risk factor for anemia. Anemia, which may result in weakened immune response, is also a risk factor for other infections to easily establish themselves. This was clarified in the revised manuscript**
