# Supplementary material for: Clinical and laboratory presentation of first-time antenatal care visits of pregnant women in Ghana, a hospital-based study
Source: PLoS One. 2023 Jan 4;18(1):e0280031. doi: 10.1371/journal.pone.0280031 (PMC9812315; doi:10.1371/journal.pone.0280031)
Supplement: Attachment — Submitted filename: Response to Reviewers.docx [file pone.0280031.s003.docx]

November 11^th^, 2022

PLOS ONE

Dear editor,

Thank you for the opportunity to revise our manuscript titled “Clinical and laboratory presentation of first-time antenatal care visits of pregnant women in Ghana, a hospital-based study” with reference number “PONE-D-21-22935”.

We find the reviewer(s) comments helpful and have therefore revised the manuscript with the reviewer’s comments to the authors. We have provided a response (highlighted) to the comments in the detailed document attached.

Thank you,

Maxwell Akonde

akondemaxwell@gmail.com

Response to reviewer’s comments

I would emphasize that the reduced study time period unable the authors to describe the seasonality variability in some disease, especially malaria, and that should be mentioned in the limitations of the study. We have addressed malaria seasonality by discussing this in the limitations.
In line 92, there should be an information concerning the hemoglobinometer used in the study. This is addressed. The hemoglobinometer used was the URIT12® from URIT medical electronics Co. Ltd, Guangxi, China
Line 94, it misses “into less than 11.5g/dl and (more than) 11.5g/dl.” This has been addressed. The categories were less than 11.5g/dl and 11.5/dl or more
Line 110 and 111. The G&PD activity method should be explained, and the reference values for G6PD deficiency should be mentioned. The methemoglobin reduction method has been explained. The methemoglobin reduction method, where through the action of nitrite, hemoglobin is oxidized to methemoglobin and in the presence of methylene blue, methemoglobin is reduced back to hemoglobin through the oxidative pathway, was used. G6PD is the enzyme needed for the reduction of methemoglobin and in its absence the solution remains brown. The test was performed by putting 2ml of participants blood in three tubes labeled “positive”, “negative”, and “test”. In the tubes labeled test and positive, 0.1ml of sodium nitrite-glucose solution was added and methylene blue additionally added to the tube labeled test and the solutions mixed gently. No reagent was added to the tube labeled negative. All the tubes were incubated for 3 hours, and then 0.1ml of the solutions of each of the tubes were taken into newly labeled tubes and the volumes were made up to 10ml using normal saline. The color and clear nature of tubes labeled test were compared to the positive and negative. The test tubes that were clear red and similar to the tubes labeled negative are reported as no defect, those that were brown and similar to the tubes labeled positive were reported as full defect and those intermediates were reported partial defect.
Line 131. Authors have include HBB C allele as sickling allele, but it is not classified as a sickling allele, it indeed protects from sickling in SC patients. In that sense I would suggest to refer just 10.4 % sickling. The authors does not refer which method they use to test sickling, or if they are using electrophoresis result to consider sickling. This has been addressed. The sodium metabisulfite test was used for the sickling status. It is true HB C does offer some protection for SC, however it still carries the sickle cells and hence sickles under the metabisulphite solution as well the HB S separates out on the electrophoresis. Thus HB SC test positive for sickling.
Table 1 -I would suggest to include max and min values in all variables. There are no max and min for the categorical variables on Table 1. With respect to the numerical variables (weight, age, blood pressure and hemoglobin, these were normally distributed and hence, were presented as means and standard deviations. A max and min (range) would pass for skewed data where medians are presented.
There is no information concerning the existence (or not) of multiple infection (or mixed infections) in any pregnant women. Multiple infection was not a subject of question in this study. This is strengthened by the fact that infection rates were generally lower and hence statistics on multiple infections will produce very smaller numbers
There is no analysis of G6PD def or HBB genotype as a risk factor for infection or other laboratory parameter alteration. This scope of the manuscript is descriptive as noted by the reviewer. We therefore did not assess how other variables predict or act as risk factors for infection.
Line 152 and 153 – The comparation is not informative. Authors should determine the frequency in each classes (22/26 and 157/162) and compare those results. These sentence should be rewrite. This has been addressed. I have referenced the percentages in the sentences as suggested.
There are any result or data in pregnancy complications? An its association with G6PD, HBB, Infections etc. The study did not collect data on pregnancy complications except for the data presented, and hence relationship between G6PD, HBB, infections, etc could not be studied.
Discussion
Line 175. In the sentence “Overall, there have been improvement in certain” there is no information in which parameters and compared to what values or periods or regions. We have addressed this. I provided a reference for the situation two decades ago and what we have from our data.
Line 179 – “This is more than the average of 56% prevalence of anemia in developing countries” this is to the general population? And for pregnant women? This is the percentage of anemia in pregnant living in developing countries as estimated by the WHO in the reference.
Line 189. There is lacking information if the pregnant women from the study use Vitamin supplements. Even though the Ghana health service recommends supplements, health facilities do not collect data on these supplements and hence information on vitamins and other nutrients supplements was unavailable for this study.
Lijne 209 – 211 . Please clarify the sentence. Does the authors refers to preventive therapeutics? The sentence refers to the malaria control programs such as indoor residual spraying, and use of insecticides treated bed nets that are targeted at preventing malaria. The sentence advocates the need to do more to ensure pregnant women are benefiting and adhering to these measures.
Is missing discussion concerning Mixed infections, influence of S allele and C allele, G6PD deficiency. The study did not present data on mixed infections as previously explained.
Limitations – Please clarify sentence in line 231-232 Because the study design is cross-sectional, the study is limited to determining whether control programs as stated earlier in the discussion caused the declines. This may be a topic for subsequent research.
Include as limitation : small sample size, just 4 months of study, doesn’t covering malaria seasonality or food availability We have included sample size and malaria seasonality as a limitation, however food availability and assessing that was not within the scope of this study. Food availability has not been a challenge in Ghana since the 1980’s.
Fig 2 -it should be represented in % by hemoglobin category and not absolute numbers. We have updated figure 2 to reflect percentages
