# Supplementary material for: Clinical and laboratory presentation of first-time antenatal care visits of pregnant women in Ghana, a hospital-based study
Source: PLoS One. 2023 Jan 4;18(1):e0280031. doi: 10.1371/journal.pone.0280031 (PMC9812315; doi:10.1371/journal.pone.0280031)
Supplement: Attachment — Submitted filename: Response to Reviewers.docx [file pone.0280031.s004.docx]

December 6th, 2022

PLOS ONE

Dear editor,

Thank you for the opportunity to revise our manuscript titled “Clinical and laboratory presentation of first-time antenatal care visits of pregnant women in Ghana, a hospital-based study” with reference number “PONE-D-21-22935”.

We find the reviewer(s) comments helpful and have therefore revised the manuscript with the reviewer’s comments to the authors. We have provided a response (highlighted) to the comments in the detailed document attached.

Thank you,

Maxwell Akonde

akondemaxwell@gmail.com

Response to reviewer’s comments

Reviewer #3: 1. Line 40: The method for determination of G6PD status should be methaemoglobin reduction test, and not cyanmethaeglobin (which is used for hemoglobin determination): We have corrected this and elaborated on the methaemoglobin method used.

2. Methods: Please describe how clinical and laboratory data was collected. Was the data collected from electronic records or from paper folders.: This hospital had an electronic record system. We clarified that by inserting: “Access to the completely de-identified data through the hospitals electronic system was granted for the purpose of this study in November 2018.”

3. Line 90: Continuous should be "continuous variables": “Variables” is inserted

4. Line 99: Please change "malaria was performed" to "malaria testing was performed": “Testing” is inserted.

5. Line 101: It is not clear that malaria microscopy was used to confirm RDT results. Is this the normal practice? How is this done (are both tests done simultaneously or it is only RDT positive samples that are further tested with microscopy)?: Malaria microscopy is done for only RDT positive samples. We added the following sentence to clarify this: “A confirmation test for malaria parasites was done with blood film microscopy for samples that tested positive with the lateral flow technique.”

6. Line 140: Please add the sample size to the percentages, e.g. 12.8% (n/N): We strongly believe that reporting percentages and the actual counts will amount to repeating the results. We have the actual counts in the tables which makes referencing it when needed easier, however the percentages better describe the results and hence the consistency throughout the results section as seen in manuscripts published by Plos One Journal.

7. Table 1: Please change "hemoglobin electrophoresis" to "hemoglobin variants": We have changed the “electrophoresis” to “variants”.

8: Line 208: The anemia prevalence could also be compared to other African countries: We added comparative findings from Nigeria to the discussion

9. Please check that all references are complete, e.g. Ref 4,: We checked all references and ensured they were complete. We revised Reference 4 to make it complete.
